# Computational repurposing of polyphenols for anti-Mpoxviral activity

**DOI:** 10.1007/s40203-025-00345-1

**Published:** 2025-04-17

**Authors:** Rishi Vachaspathy Astakala, Gagan Preet, Ahlam Haj Hasan, Ria Desai, Meshari Alfurayh, Rainer Ebel, Marcel Jaspars

**Affiliations:** 1 Marine Biodiscovery Centre, Department of Chemistry, University of Aberdeen, Scotland, AB24 3FX UK; 2 The Medicinal Chemistry and Pharmacognosy Department, College of Pharmacy, Jordan University of Science and Technology, Irbid, 22110 Jordan

**Keywords:** Mpox, *Orthopoxvirus*, Polyphenols, Virtual screening, Molecular dynamics, Pharmacophore, Structure–activity relationship

## Abstract

Mpox is a globally prevalent disease that has triggered multiple epidemics over the past few decades, leading to moderate rates of hospitalisation and mortality. Recently, it has re-emerged in several countries, including the Democratic Republic of Congo, and appears to be spreading at an unprecedented pace. The disease is caused by zoonotic double-stranded DNA viruses. Due to its similarities with smallpox, distinguishing between the two can be challenging, though the smallpox vaccine typically provides immunity against Mpox. At the time of writing, no approved treatment for Mpox exists; however, several promising candidates have demonstrated the ability to inhibit viral replication, including resveratrol—a polyphenolic compound found in red wine. This study employs molecular docking and molecular dynamics simulations to assess the effectiveness and stability of nine resveratrol analogues. Additionally, 2D and 3D pharmacophore models were developed for the highest-ranked docked compounds, leading to a composite pharmacophore. A structure–activity relationship analysis was also conducted using these top-performing compounds. The findings suggest that two compounds— (**9**) [1,1'-biphenyl]-3,4',5-triol and (**11**) {3-hydroxy-5-[2-(4-hydroxyphenyl)ethenyl]phenyl}oxidanesulfonic acid—exhibit strong binding affinity, with compound 11 potentially forming a stable complex with the thymidylate kinase of the vaccinia virus.

**Graphical abstract:**

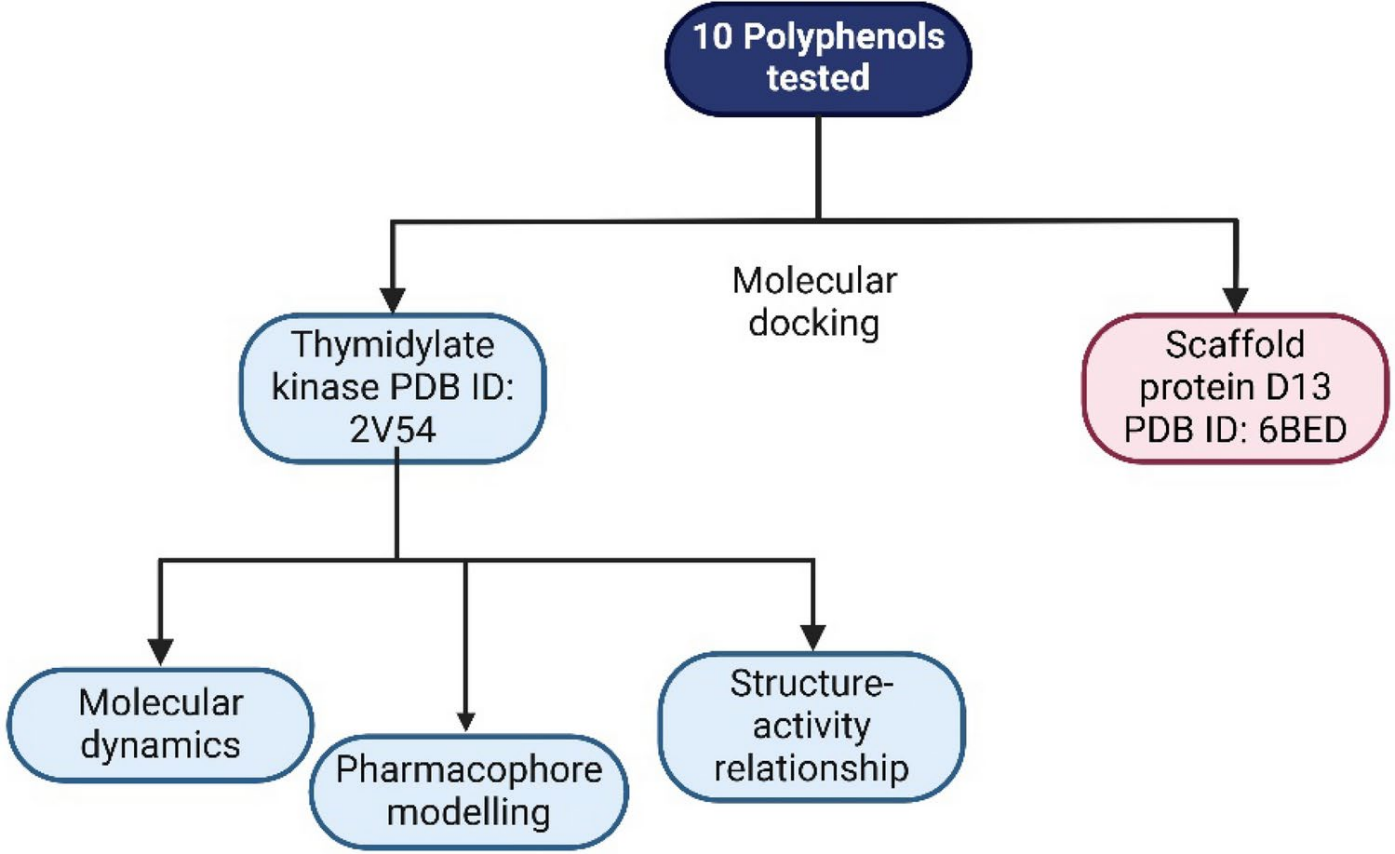

**Supplementary Information:**

The online version contains supplementary material available at 10.1007/s40203-025-00345-1.

## Introduction

Mpox is a viral illness caused by the mpox virus (MPXV), a double-stranded DNA virus from the Poxviridae family. Initially identified in the Democratic Republic of Congo (DRC), MPXV is now endemic in several Central and West African countries, including Ghana, Benin, and Cameroon. However, over the past two decades, there have been two outbreaks beyond this region, notably in Sudan in 2005, which resulted in 49 reported cases (Formenty et al. 2010). More recently, between 2022 and November 2023, outbreaks were documented in several other countries, including India, Singapore, the U.K., and the United States, with a total of 92,167 cases reported globally (2022 Monkeypox Outbreak Global Map| Monkeypox| Poxvirus| CDC. Accessed 2 December 2022) (Bunge et al. 2022). However, as of 29 March 2024, the DRC has reported 18,922 cases, with 1007 deaths and 488 cases reported in 2024 alone (European Centre for Disease Prevention and Control, Accessed 28 May 2024).

The *Poxviridae* family also includes the smallpox virus, vaccinia virus, and variola virus, all classified under the genus *Orthopoxvirus*. This genus is part of the *Chordopoxviridae* subfamily within *Poxviridae*, which primarily infects vertebrates (Cheema et al. 2022). MPXV exhibits symptoms similar to smallpox, which led to its initial misidentification as a distinct disease. It was first discovered in monkeys, with its recognition in humans occurring only after the 1970s. Smallpox, one of the deadliest diseases known to humans, has a mortality rate of 30% (Brown and Leggat 2016) and high virulence. MPXV shares high virulence and much of its clinical presentation with the variola virus, which causes smallpox. Symptoms of monkeypox include fever (ranging from 38.5 to 40.5 °C), rash, malaise, headache, and characteristic tough, umbilicated, well-defined lesions. The virus has an incubation period of 4–14 days, and the symptoms can last up to 4 weeks. A key feature that distinguishes monkeypox from smallpox is the presence of tender lymphadenopathy in the maxillary, cervical, and inguinal regions (Cheema et al. 2022), which is absent in smallpox. The presence of the lymphadenopathy itself, however, may indicate a robust immune system recognition and response to the virus (McCollum and Damon 2014). The clinical presentation of the virus extends beyond the symptoms previously mentioned. It has also been associated with neurological issues such as seizures, myalgias, and encephalitis, which may result from the invasion of astrocytes and the activation of the inflammasome, leading to pyroptosis (Miranzadeh Mahabadi et al. 2024). Ocular symptoms, including conjunctivitis, skin lesions, corneal ulcers, and uveitis, have also been observed in some RT-PCR-positive cases (Androudi et al. 2024; Curi et al. 2024). Individuals who were previously vaccinated against smallpox often exhibit resistance to, or even immunity against, mpox (Moore et al. 2022).

The virus is transmitted from animals to humans through direct contact and biting and scratching. While many mammals are susceptible to the virus, the original host remains unidentified. In humans, it spreads through contact with the lesions of an infected individual or their belongings, such as clothing and bedding, as well as through respiratory droplets, typically requiring prolonged face-to-face interaction (Brown and Leggat 2016). These cases were traced to infected imported mammals from Ghana or people travelling from countries where it is endemic (Brown and Leggat 2016).

Historically, case fatality rates have varied significantly between viral clades: the Central African clade has an average fatality rate of 10.6%, whereas the West African clade averages 3.6%. The 2022 outbreak had an estimated fatality rate of 0.03%, potentially due to factors such as patient age, viral mutations leading to a less severe infection, or selection bias. In regions with historically high fatality rates, underreporting may occur as individuals in severely affected areas often seek medical care only when critically ill (Vogel 2022). In the year 2023, 12,569 suspected cases were recorded, with a suspected case fatality rate of 4.6% in the DRC alone, the highest number of cases ever reported in a year (WHO 2023). Other means of transmission, including sexual contact, which before April 2023 had not been documented for the Central African clade, and other unknown modes have left us with an incomplete understanding of the dynamics of the outbreak, which has raised further concerns (WHO 2023). A recent study has found mutations in the viral genome potentially explaining the increased transmission rate (Monzón et al. 2024). According to one study, the rate of mutation of MPXV has been observed to increase since 2017, which might suggest sustained human-to-human transmission (O'Toole et al. 2023).

It is, therefore, imperative to address this rapidly spreading viral disease swiftly and effectively.

**Fig. 1:**
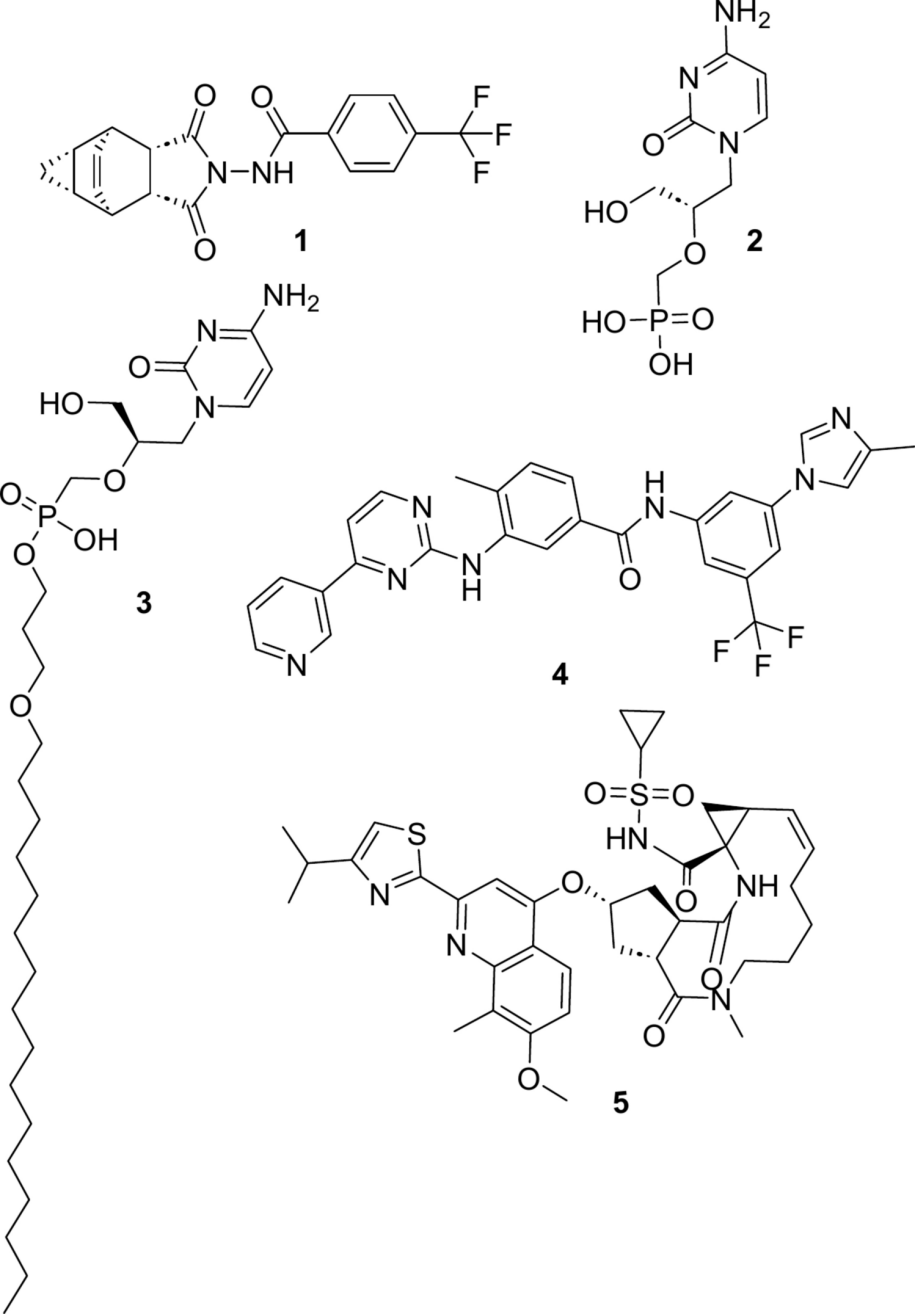
The structures of anti-mpox compounds recommended by the American CDC: tecovirimat (**1**), cidofovir (**2**), and brincidofovir (**3**), along with potential candidates for targeting viral replication, nilotinib (**4**) and structural integrity, simeprevir (**5**) discovered by Lam et al. 2022

Diagnosing mpox is often done through an individual's inspection of the symptoms. However, the gold standards for diagnosing mpox are PCR testing, electron microscopy, and virus isolation from a vesicular swab (Li et al. 2006; Neubauer et al. 1998; Panning et al. 2004). In a study conducted at the University at Pompeu Fabra (UPF), Barcelona, Spain, an intradermal vaccine, JYNNEOS, has been found to induce an immune response even in individuals infected with HIV-1 with susceptibility towards MPXV (Sisteré‐Oró et al., 2024) and has been approved for usage for the prevention of mpox in the USA (Centers for Disease Control and Prevention & National Center for Emerging and Zoonotic Infectious Diseases (NCEZID), 2024). The ACAM2000 vaccine, which was initially used for smallpox, has the potential to be made available for mpox under a new protocol (Centers for Disease Control and Prevention & National Center for Emerging and Zoonotic Infectious Diseases (NCEZID), 2024). The US Centers for Disease Control (CDC), in interim clinical guidance, has recommended the usage of drugs typically used against smallpox, such as tecovirimat (**1**) (Fig. 1), cidofovir (**2**), brincidofovir (**3**) and vaccinia immunoglobulin as countermeasures against mpox cases where the patient is severely immunocompromised (CDC Monkeypox Interim Clinical Guidance 2024). Further studies are being conducted on the efficacy of tecovirimat against monkeypox.

A computational study to repurpose drugs targeting various proteins crucial for viral replication and structural integrity discovered several potential candidates, including nilotinib (**4**) as a DNA ligase inhibitor and simeprevir (**5**) as a compound that inhibits the viral capsid, a protein enclosing the genetic material of the virus (Lam et al. 2022). Computational studies designing vaccines based on multiple epitopes have also been conducted. These studies rely on phylogenetic analysis followed by lymphocyte prediction, antigenicity prediction followed by stability studies to produce a hypothetical multi-epitope-based vaccine (Aziz et al. 2022; Hayat et al. 2022). In a previous paper, we studied the antiviral effect of structural analogues of the anthraquinone derivative mitoxantrone (Preet et al. 2022).

As of writing this study, there is no approved treatment for mpox, but several promising candidates for lead compounds have been shown to suppress poxviral replication. Resveratrol (**RVR**) is one such candidate (Cao et al. 2017), Which suppresses vaccinia virus replication with an IC50 of 4.72 μM. The vaccinia virus is often called the prototypical member of the *Poxviridae* family. Resveratrol (3,5,4'trihydroxy-trans-stilbene) is a stilbenoid compound, a class of polyphenols plants produce as a stress response. It is found in various fruits, such as grapes and blueberries (Catalgol et al. 2012). This study presents the docking study of 9 polyphenols with structures similar to resveratrol (**7**–**15**) selected from the COCONUT database (Sorokina et al. 2021) based on the Tanimoto similarity score.

## Results and discussion

### Molecular docking evaluation

The antiviral activity of 9 polyphenolic compounds (SMILES included in Table S1) was studied using molecular docking (results shown in Table 1). Resveratrol was the standard because it was shown to be inhibiting virus replication (Cao et al. 2017). The COCONUT database (Sorokina et al. 2021) was used to search for structural analogues based on the Tanimoto similarity index, from which nine compounds with a greater than 90% rating were chosen for this study. The compounds were filtered based on their retention of the stilbenoid structural moiety. This study uses two proteins that play an essential role in poxvirus replication, the first of which is the thymidylate kinase of the vaccinia virus (PDB ID: 2V54) with a 2.40 Å resolution (Caillat et al. 2008). The thymidylate kinase is a protein involved in the DNA synthesis of the virus, with which resveratrol is known to interfere (Cao et al. 2017). It is an oft-targeted protein for anti-orthopoxviral drug development (Prichard and Kern 2012) since it is phylogenetically conserved in orthopoxviruses (Abdizadeh 2023). The second protein used in this study is the scaffold protein D13 of the vaccinia virus (PDB ID: 6BED) with a 2.75 Å resolution (Garriga et al. 2018). A critical difference between poxviruses and other enveloped viruses is that poxviruses rely on precursors of the cytoplasmic membrane to form the viral envelope onto a scaffold formed by the D13 protein (Garriga et al. 2018). The co-crystallised ligand in the crystal structure of the thymidylate kinase of the vaccinia virus (PDB ID: 2V54), thymidine diphosphate (TDP), was used to validate the docking protocol (Sect. 3.2.1).

**Table 1:** Docking score of the ten compounds against both the proteins. The highlighted compounds are the best-docked compounds

Compound (COCONUT ID)	Docking score (Kcal/mol)	
	2V54 (*Thymidylate Kinase*)	6BED (Scaffold protein D13)
	− 7.8	− 6.9
	− 7.7	− 6.5
	− 7.8	− 6.9
	− 8.9	− 6.9
	− 8.7	− 7.2
	− 9.5	− 8.1
	− 8.1	− 7.6
	− 7.6	− 6.8
	− 7.7	− 7.2
	− 8.3	− 7.2

Each compound's docking pose was compared to the standard resveratrol (**RVR**). Based on their docking scores and interactions with the proteins, compounds **9, 10, 11, 12,** and** 15** were found to have the leading docking scores (Table 1).

For the *thymidylate kinase* of the vaccinia virus (PDB ID: 2V54) the residues Pro39(B), Arg93(B), Tyr101(B), Ser97(B), Gly98(B), Phe68(B), Leu53(B) are interacting hydrophobically with all five compounds except for Arg93(B) which also interacts through a hydrogen bond to compound **11** at 3.16 Å. Compound 6 also interacts through hydrogen bonding with Arg41(B), Lys17(B), and Asn37(B) at 3.17, 3.02, and 3.23 Å, respectively.

**Fig. 2:**
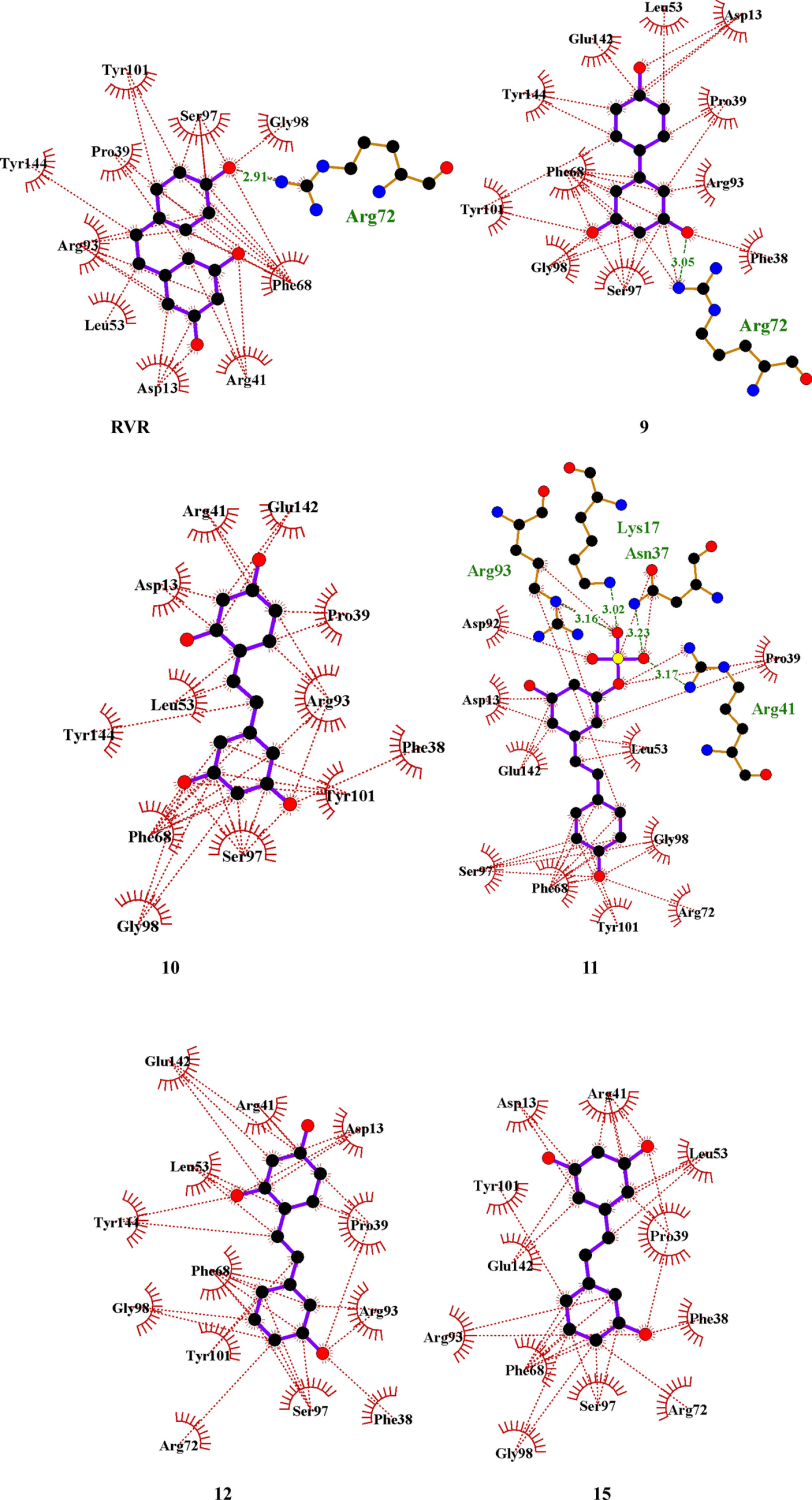
Ligplots showing the top-docked compounds' interactions with the vaccinia virus's *thymidylate kinase* (PDB ID: 2V54). Purple lines indicate polyphenol ligand bonds; orange lines indicate non-ligand bonds; green dotted lines indicate hydrogen bonds with distances in Å; red dotted lines show hydrophobic interactions; red circles indicate oxygen atoms; blue circles indicate nitrogen atoms; black circles indicate carbon atoms; radial lines indicate non-ligand residues involved in hydrophobic contact(s)

The residues Phe38(B) are similar in all aspects to the residue Tyr144(B) except for one: they interact hydrophobically with all five compounds except for compound **11** and the standard **RVR,** whereas the residue Tyr144(B) interacts with **RVR** as well. Residues Arg72(B) and Asp92(B) interact hydrophobically with compound **11,** while Arg72(B) also interacts with **RVR** and compound **9** hydrophobically and through a hydrogen bond. The Ligplots with the interactions can be found in Fig. 2.

For the Scaffold protein D13 of the vaccinia virus (PDB ID: 6BED), the residues Lys429(B), Asn117(B), Ile120(B), Tyr116(B) and Asn472(B) interact hydrophobically with compounds **9, 10, 12** and the standard **RVR.** At the same time**,** the residue Ile120(B) also interacts with compound **10,** and the residue Asn472(B) interacts with compound **15** through a hydrogen bond at 2.94 Å**.** The residues Gly473(B) and Lys434(B) interact solely with **RVR** through hydrophobic interactions, and the residue Tyr 283(B) interacts solely with **RVR** through a hydrogen bond at 2.83 Å.

**Fig. 3:**
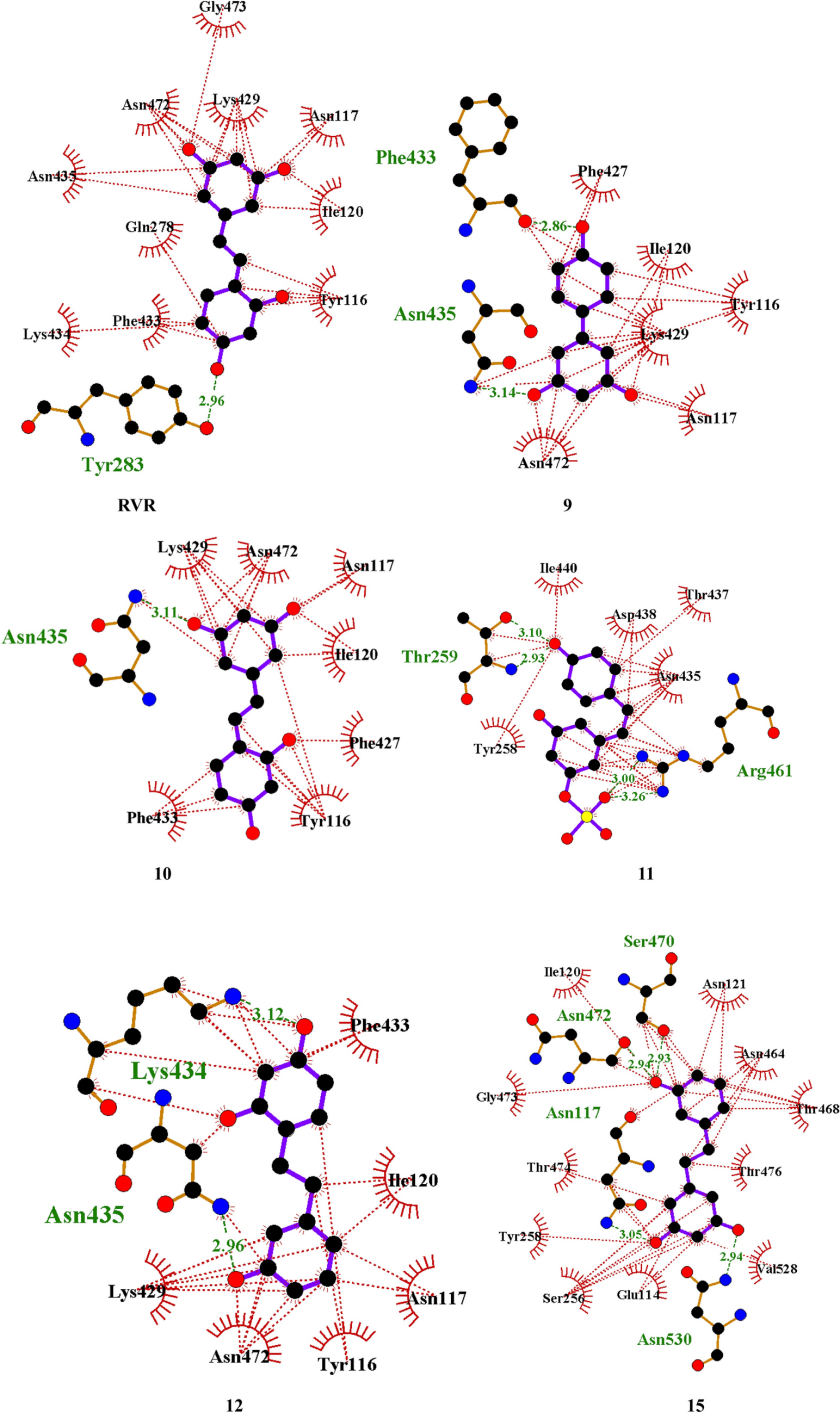
Ligplots of the top docked compounds interacting with the scaffold protein D13 of the vaccinia virus (PDB ID: 6BED). Purple lines indicate polyphenol ligand bonds; orange lines indicate non-ligand bonds; green dotted lines indicate hydrogen bonds with distances in Å; red dotted lines show hydrophobic interactions; red circles indicate oxygen atoms; blue circles indicate nitrogen atoms; black circles indicate carbon atoms; radial lines indicate non-ligand residues involved in hydrophobic contact(s)

The residues Ile440(B), Asp438(B), Thr437(B), and Tyr258(B) interact solely with compound **11** through hydrophobic interactions, and the residues Arg461(B) and Thr259(B) interact solely with compound **11** via hydrogen bonding at 3.26 and 3.10 Å. The residue Phe433(B) interacts solely with compound **12** through a hydrophobic interaction. The residues Asn121(B), Asn464(B), Thr468(B), Thr476(B), Val528(B), Glu114(B), Ser256(B), Thr474(B), and Gly473(B) interact solely with compound **15** through hydrophobic interactions and the residues Ser470(B), Asn530(B) and Asn117(B) interact solely with compound **15** through hydrogen bonds. The Ligplots with the interactions can be found in Fig. 3.

### Pharmacophore evaluation

**Fig. 4:**
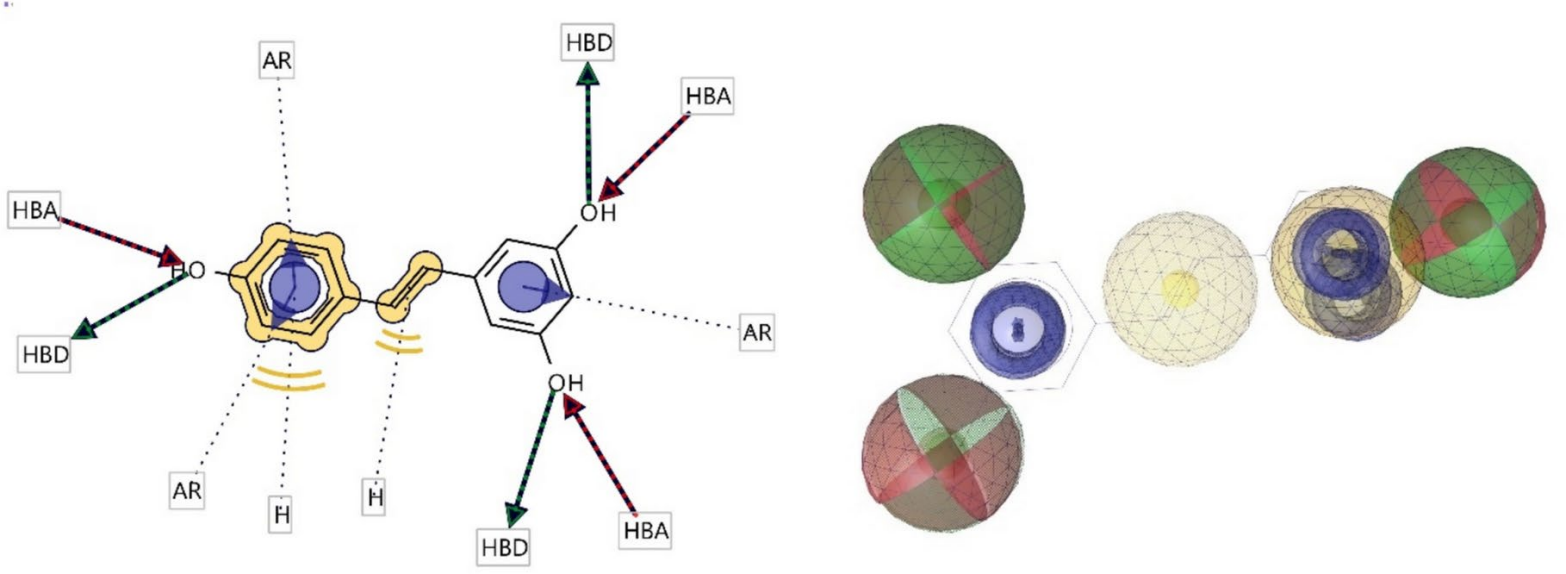
2D and 3D representations of the pharmacophoric features of **RVR** (Red shows Hydrogen Bond Acceptors; green shows Hydrogen Bond Donors; Yellow shows Hydrogens; Purple shows Aromatic Rings.)

**Fig. 5:**
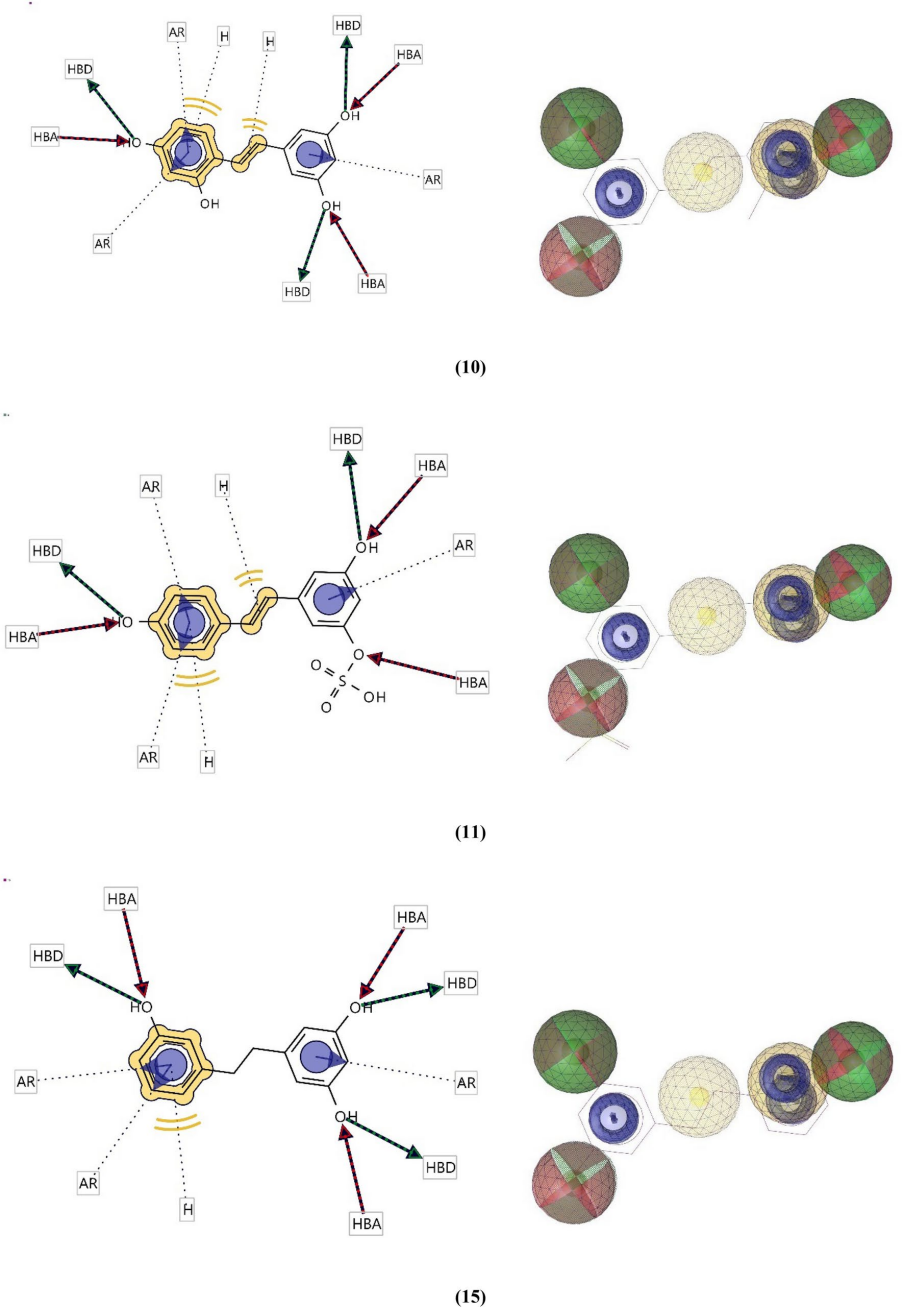
2D and 3D representations of the pharmacophoric features of compounds **10**, **11**, and **15** which are the top docked compounds against both proteins. (Red shows Hydrogen Bond Acceptors; green shows Hydrogen Bond Donors; Yellow shows Hydrogens; Purple shows Aromatic Rings.)

**Fig. 6:**
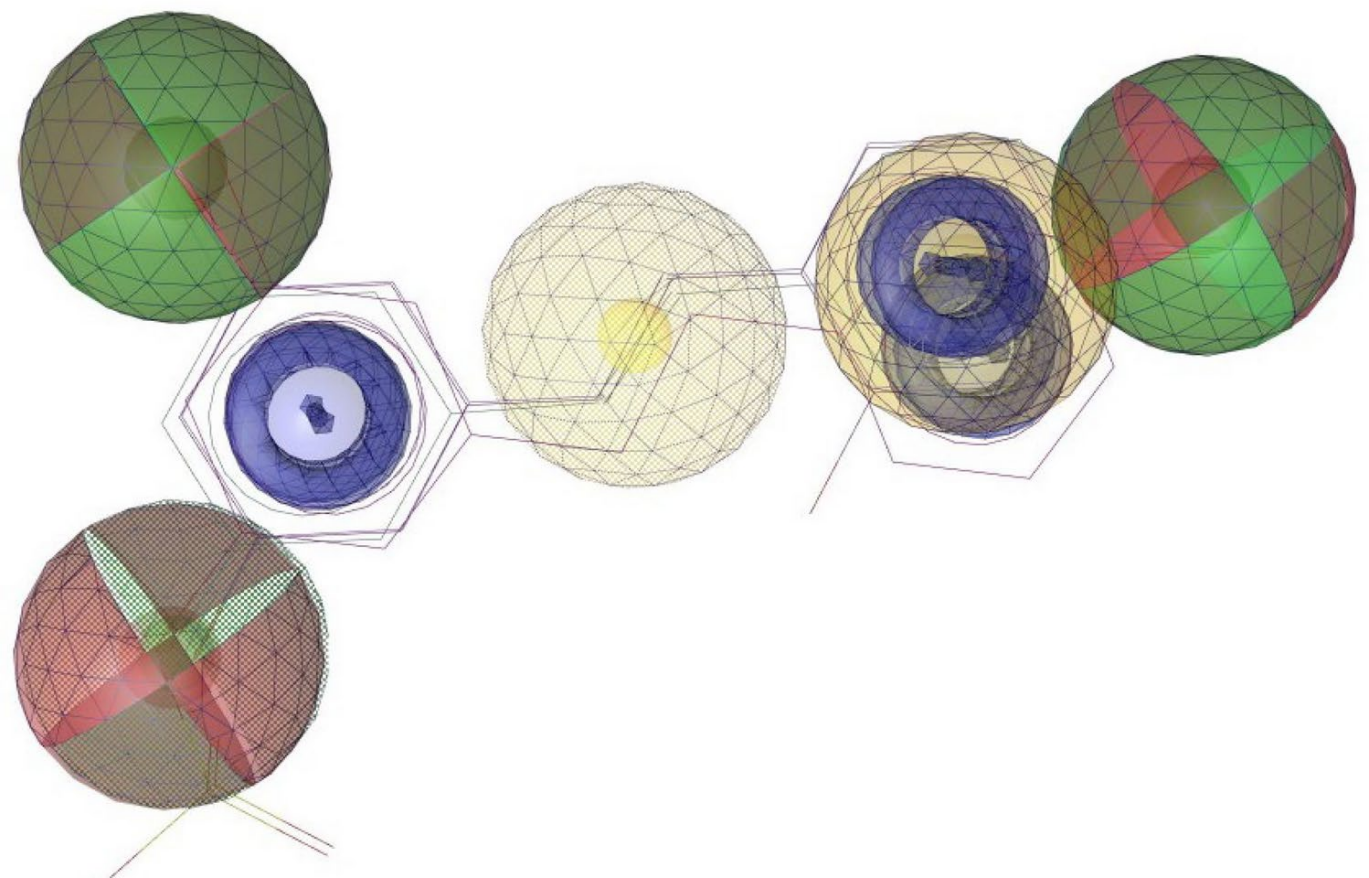
Composite 3D pharmacophore based on the top-docked compounds against both proteins. Colour codes are analogous to those in Fig. 5: Red shows Hydrogen Bond Acceptors; green shows Hydrogen Bond Donors; Yellow shows Hydrogens; Purple shows Aromatic Rings

Relying on the lowest energy conformers of **RVR** and the highest docked compounds for both proteins **10, 11,** and** 15,** a ligand-based pharmacophore showed four key features. The features included hydrogen bond acceptors (HBA), hydrogen bond donors (HBD), hydrophobic interactions(H), and aromatic rings (AR). The 2D and 3D representations of the pharmacophoric features of each compound are shown in Figs. 4 and 5. Each of the compounds is composed of individual pharmacophoric features, and from these individual features, a composite pharmacophore was generated, which had a score of 0.91 (shown in Fig. 6).

### Molecular dynamics evaluation

The stability of *thymidylate kinase* from the vaccinia virus, selected for its high docking scores with the tested compounds, was simulated for 200 ns in complex with **RVR** (the standard) as well as compounds **11** and **9**—the two highest-ranked compounds with docking scores of − 9.5 and − 8.9 kcal/mol, respectively. Priority was given to this protein due to the time-intensive nature of the simulations.

**Fig. 7:**
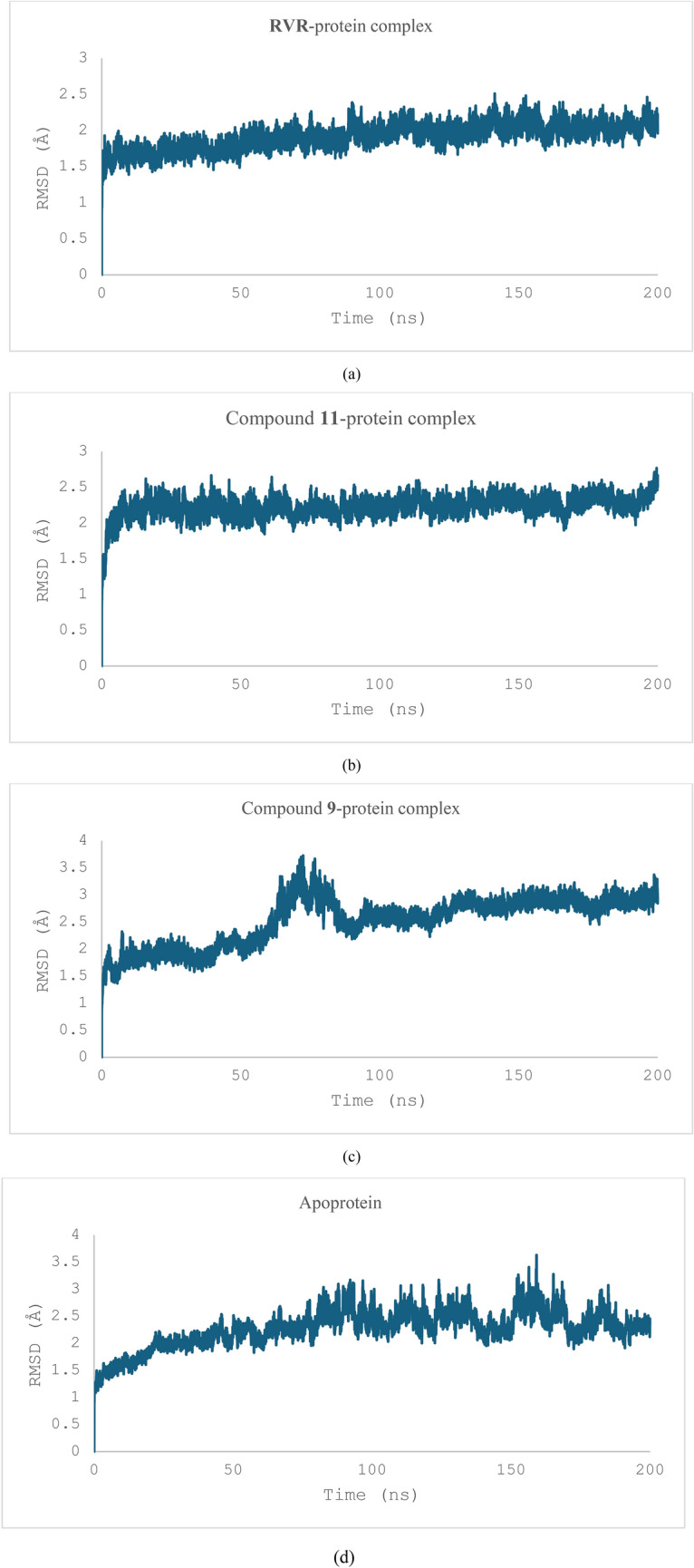
The RMSD of the α carbons of the three ligand–protein complexes: **RVR**-protein complex (**a**), Compound **11**-protein complex (**b**), compound **9**-protein complex (**c**), and that of the apoprotein (**d**) during the simulation**.** Figure 9(c) shows that the RMSD of the compound **9**-protein complex increases between 60 and 90 ns of the simulation, whereas those of the RVR-protein complex and compound **11**-protein complex appear stable throughout

Root mean square deviation (RMSD) and root mean square fluctuation (RMSF) were analysed to assess the system's behaviour. RMSD reflects overall system stability, while RMSF indicates molecular flexibility. The average RMSD for the C_α_ atoms in the RVR-protein complex was 1.92 ± 0.18 Å. In comparison, complexes with compound **11** and compound **9** had RMSD values of 2.23 ± 0.15 Å and 2.53 ± 0.45 Å, respectively. The stability of the unbound (apoprotein) thymidylate kinase was also simulated, yielding an RMSD of 2.28 ± 0.35 Å (Fig. 7d).

During the simulations, the complexes with **RVR** (Fig. 7a) and compound **11** (Fig. 7b) remained stable, whereas the compound **9** complex exhibited significant deviation between 60 and 90 ns (Fig. 7c).

**Fig. 8:**
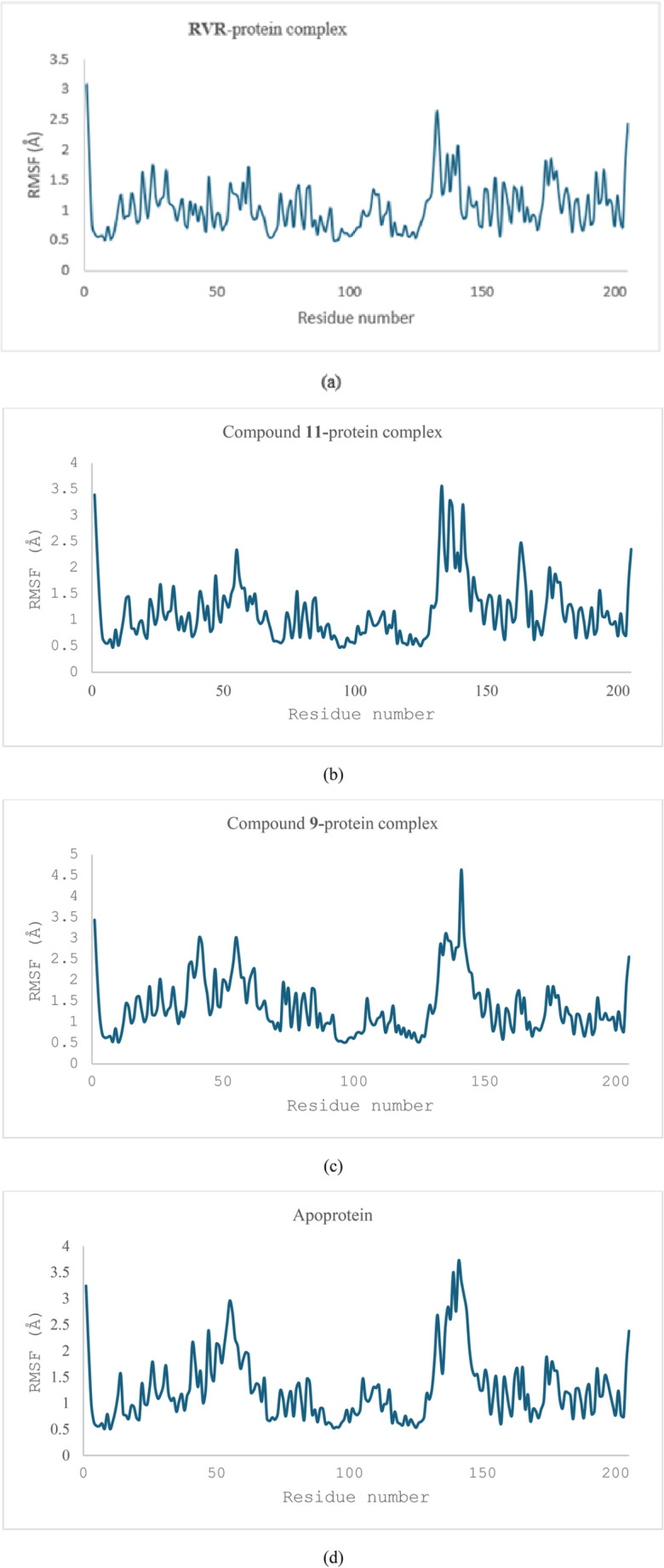
The RMSF of the three ligand–protein complexes: **RVR**-protein complex (**a**), compound **11-**protein complex (**b**), compound **9**-protein complex (**c**), and that of the apoprotein (**d**). This figure compares the flexibility of the **RVR**-protein, compound **11**-protein, and compound **9**-protein complexes, indicating their variability

The complexes showed slight variation in the RMSF, showing that **RVR** and compound **11** form relatively stable complexes, with the compound **9** complex showing a relatively high fluctuation. The RMSF values (Fig. 8) of the residues for the protein complexes with **RVR**, compounds **11** and **9** were found to be 1.04 ± 0.40, 1.15 ± 0.56, and 1.37 ± 0.67 Å respectively. The RMSF of the apoprotein was found to be 1.26 ± 0.63 Å.

**Fig. 9:**
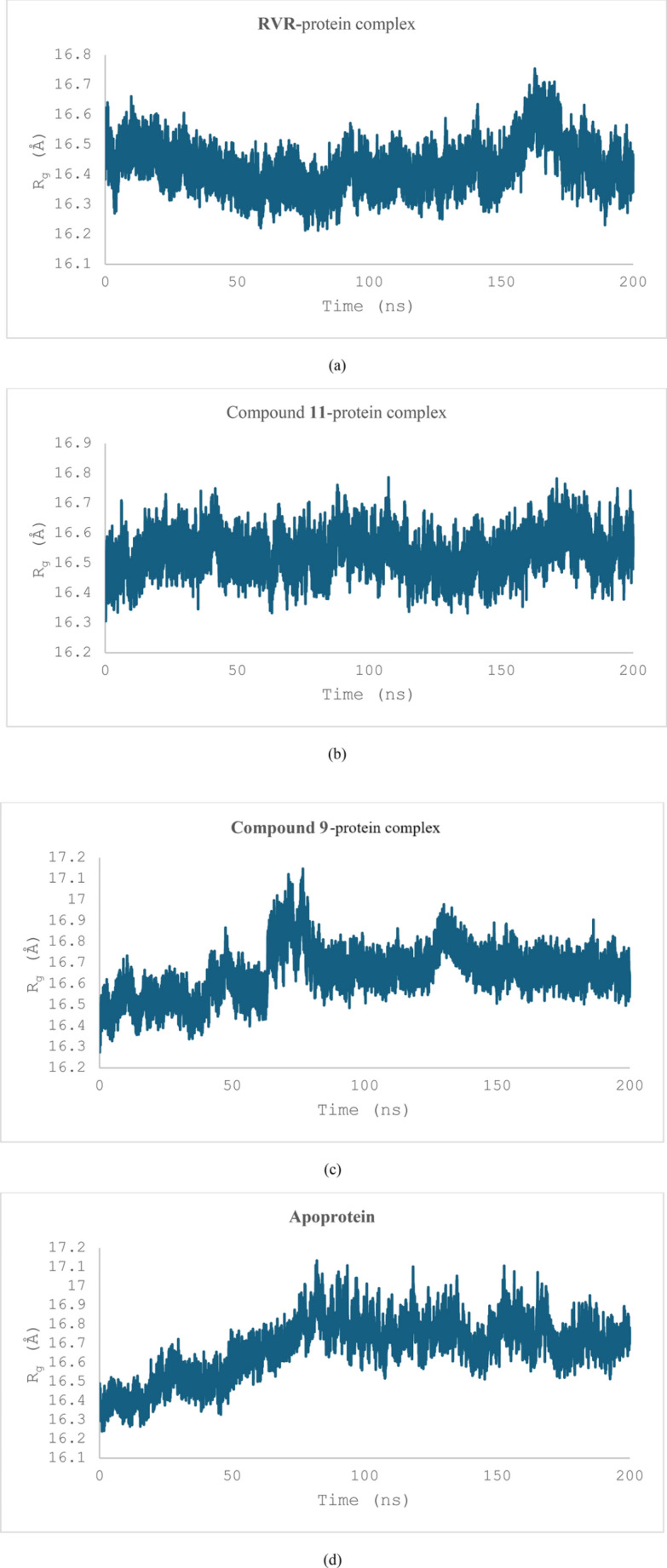
The R_g_ of the three ligand–protein complexes: **RVR**-protein complex (**a**), compound **11-**protein complex (**b**), compound **9**-protein complex (**c**), and that of the apoprotein (**d**). The R_g_ of the compound **9**-protein complex is increasing between 50 and 90 ns, which is consistent with the RMSD measurements. Another slight increase between 125 and 140 ns can be observed before it levelled off

The radius of gyration (R_g_) of the three protein–ligand complexes was also measured to check their stability (Fig. 9). The complex between **RVR** and the thymidylate kinase showed an R_g_ of 16.42 ± 0.07 Å, whereas those between compounds **11** and **9,** and the protein showed an R_g_ of 16.53 ± 0.53 Å and 16.65 ± 0.11 Å respectively. However, an important observation in the graphs for the metrics mentioned is a considerable deviation at specific points for the complex between compound **9** and the thymidylate kinase (Fig. 11c), suggesting potential instability. The R_g_ of the apoprotein was also calculated; it was found to be 16.68 ± 0.05 Å and showed a steep increase between 50 and 80 ns of the simulation before levelling off.

#### Hydrogen bond contacts

**Fig. 10:**
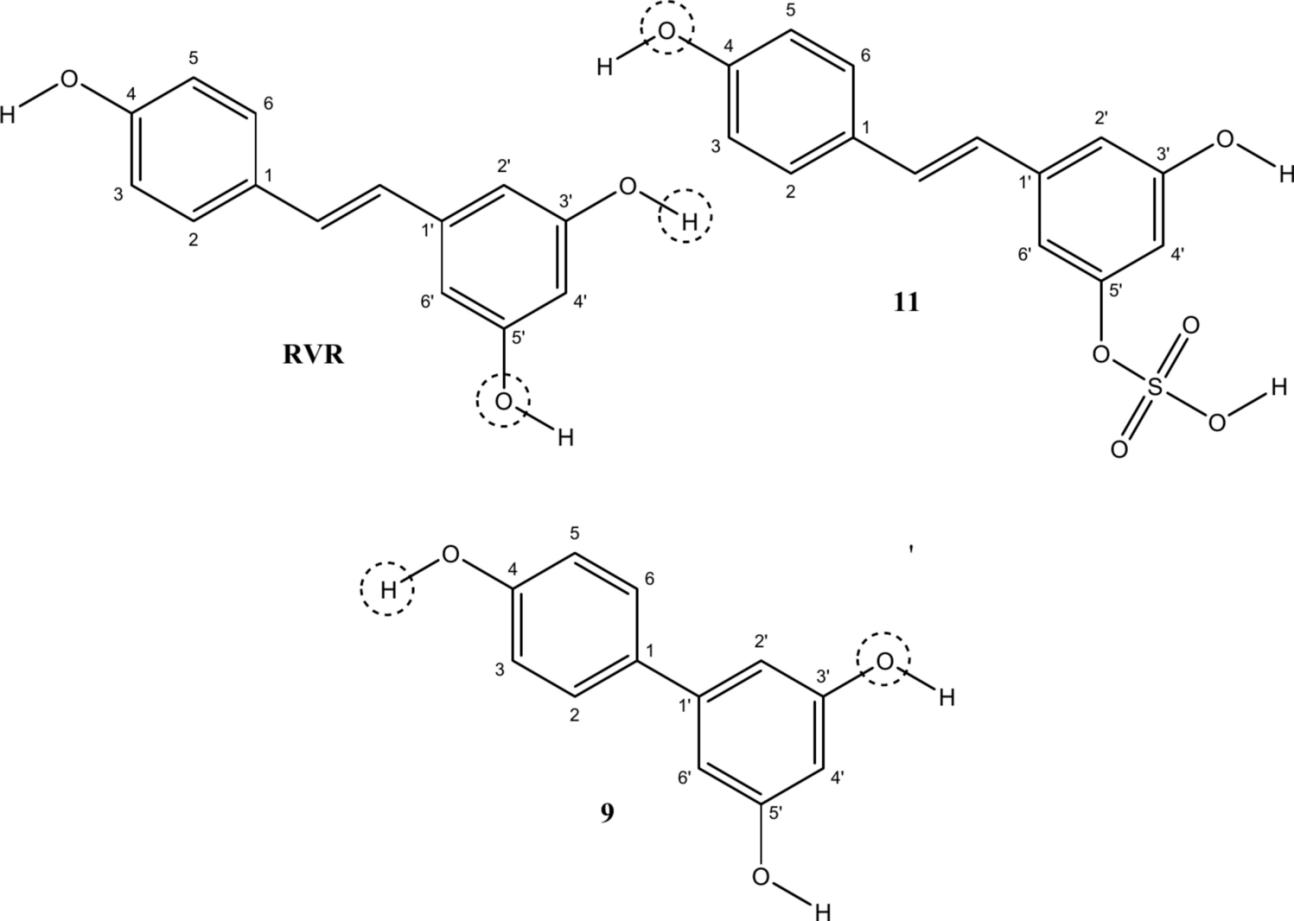
The structures of **RVR**, compounds **11**, and **9**, with positions annotated according to the standard numbering system. The atoms showing key hydrogen bonds have been highlighted for each compound

The hydrogen bond contacts of the three complexes were also calculated with **RVR** (standard), showing three moderately stable contacts with residues on the protein, namely, one between the oxygen at position 5’ ( see Fig. 10 for annotated structures) of the ligand and Hε22 of Gln150(B) (58.7% of the frames), and two between the proton at 3’-OH of the ligand and Oδ1 (57.3% of the frames), and Oδ2 (66.3% of the frames) of Asp13(B).

In contrast, the complex between compound **11** and the protein showed one moderately stable contact between the oxygen at position 4 (Fig. 10) and Hδ21 of Asn65(B), appearing for 59.8% of the frames. However, two moderately stable contacts can be seen in the complex between compound **9** and the protein between the proton at position 4-OH and Oδ1 of Asp13(B), appearing for 76.7% of the frames and another between the oxygen at position 3’ and HH12 of Arg72(B) which appears for 65.8% of the frames.

All three complexes show hydrogen bonds with water molecules appearing for various frames.

#### MM-GBSA calculations

The MM-GBSA (Molecular Mechanics-Generalised Born Surface Area) was used to calculate the binding energy of each of the top-docked ligands, and the standard with the thymidylate kinase of the vaccinia virus and the non-polar contribution was used to calculate the SASA (Solvent Accessible Surface Area) for each complex using the following formula. The values for each complex are shown in Table 2.

$$ {\text{G}}_{{{\text{sa}}}} = \gamma *{\text{SASA}} + \beta $$

$$ {\text{SASA}} = \, \left( {{\text{G}}_{{{\text{sa}}}} - \beta } \right)/\gamma $$

**Table 2:** The MM-GBSA and SASA values for each of the three complexes

Protein complexed with	ΔMM-GBSA (Kcal/mol)	Non-polar term of MM-GBSA(Kcal/mol/Å^2^)	ΔSASA(Å^2^)
RVR	− 32.35	− 33.08	− 6788
Compound 11	− 16.20	− 36.29	− 7430
Compound 9	− 19.75	− 27.59	− 5690

Where β and γ are constants whose values were assigned as 0.86 kcal/mol and 0.005 kcal/mol/ Å^2^, respectively, per the calculations done by Sitkoff et al. (1994).

#### Principal component analysis (PCA)

To analyse the conformational space explored by the three protein–ligand complexes during the simulation, we performed a Principal Component Analysis (PCA) of the trajectory.

**Fig. 11:**
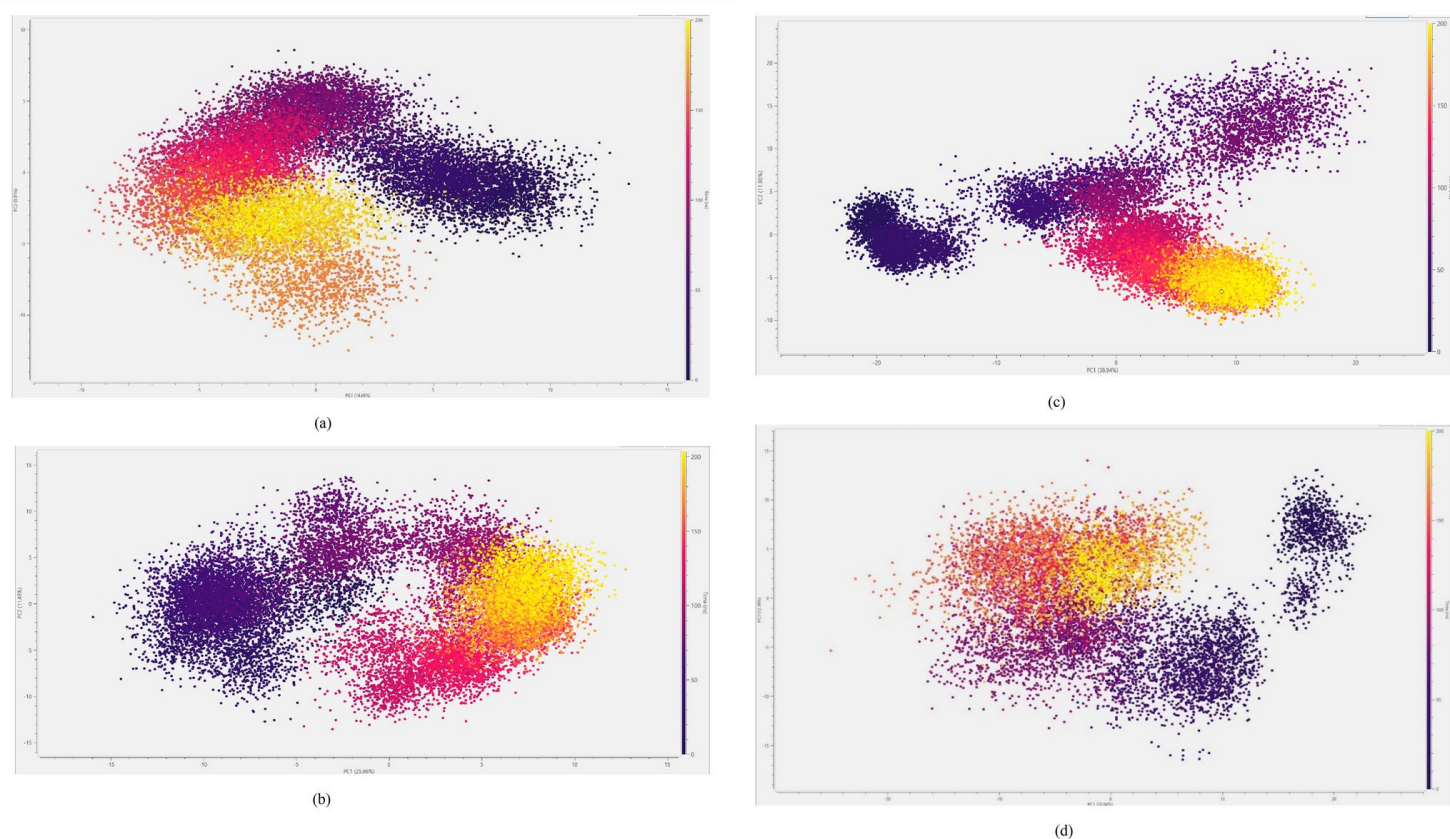
The PCA analysis of the MD trajectories of the three ligand–protein complexes: **RVR**-protein complex (**a**), compound **11-**protein complex (**b**), compound **9**-protein complex (**c**), and that of the apoprotein (**d**)

Based on the spread of the points of the graph, it can be concluded that the complexes between the protein and RVR (Fig. 11a) and compound **11** (Fig. 11b) are relatively more stable while exploring a smaller portion of the conformational space. In contrast, the complex between the protein and compound **9** is relatively less stable and explores more of the conformational space for the simulation. It can be observed from the graph of the PCA analysis for the complex between the protein and compound **9** (Fig. 11c) that the complex explores seemingly high-energy conformations between 60 and 90 ns. This observation is reflected in the RMSD graph of the same (Fig. 7c).

### ADMET property prediction

The online tool ADMETLab 2.0 (Xiong et al. 2021) was used to predict ADMET properties for **RVR** (standard), compound **11**, and compound** 9** to assess their pharmacokinetic and safety profiles. Key predicted properties are tabulated in Table 3.

**Table 3:** The predicted ADMET properties of **RVR**, compound **11**, and compound **9** and their interpretation

Property	RVR	Compound 11	Compound 9	Interpretation
Human intestinal absorption	Low	Low	Low	Predicts intestinal absorption
Blood–Brain Barrier (BBB) permeation	Low	Low	Low	Predicts CNS penetration
LogP	2.54	1.25	2.49	Predicts lipophilicity
CYP3A4 inhibitor	Very high	Moderate	Low	Predicts drug metabolism and interactions
Lipinski’s RO5	Yes	Yes	Yes	Predicts drug-likeness based on Lipinski’s rule of 5
Clearance	Moderate to high	Moderate	High	Predicts the rate of clearance
AMES toxicity	Low	Very low	Low	Predicts the mutagenic nature

The properties suggest that all three compounds have low mutagenic toxicity, a moderate to high clearance rate and in contrast, low BBB permeation and intestinal absorption.

### Structure–activity relationship (SAR)

**Fig. 12:**
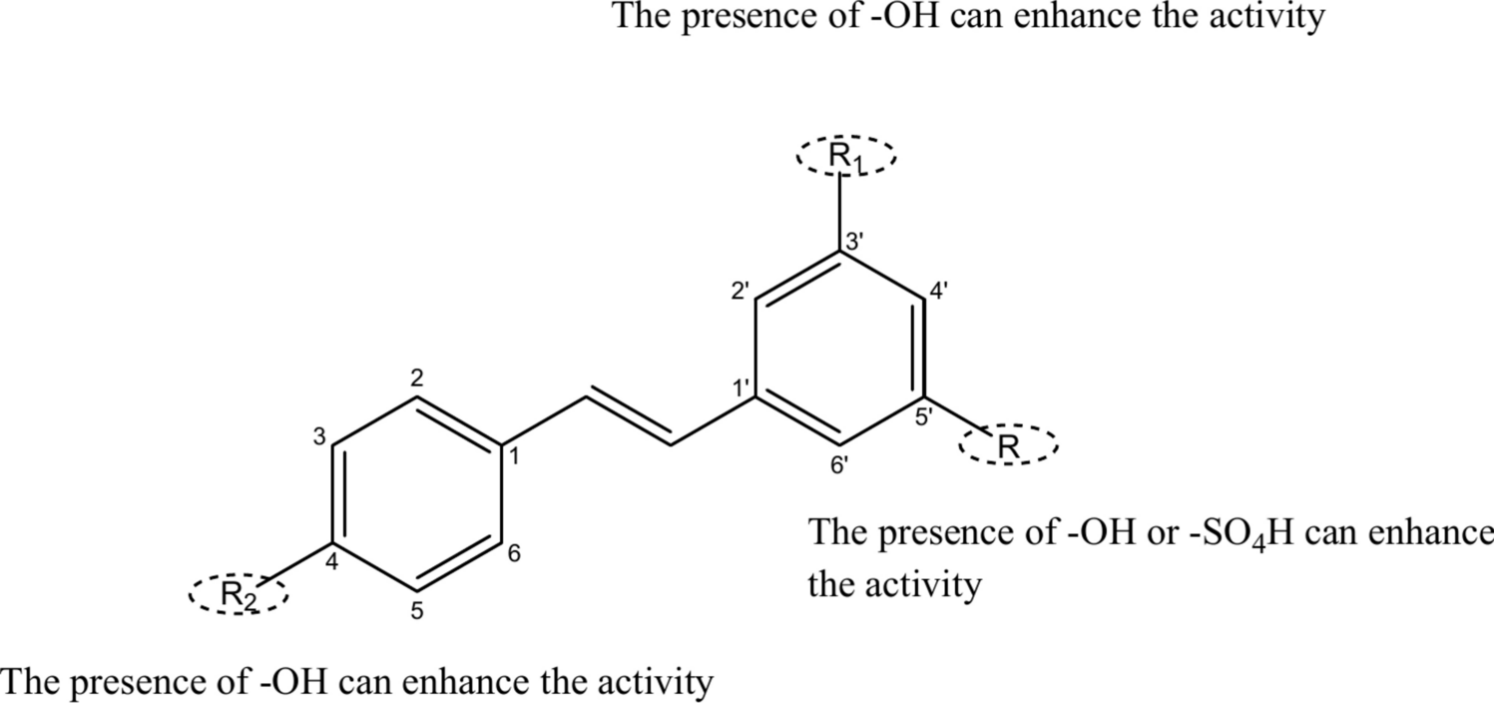
A structure–activity relationship developed for compounds studied based on the docking scores of the compounds against both proteins. The key groups are highlighted with dotted circles

Each compound's docking pose was compared to the standard resveratrol (**RVR**). Based on their docking scores and interactions with the proteins, compounds **9** and **11** were found to have the leading docking scores (Table 1). Thus, hydroxyl groups at positions C4 and C3` and hydroxyl or sulphate groups at position C5` might be essential for the activity. Compound **13** has a hydroxyl group at position C3`, but it has a lower docking score than compounds **9** and **11** because it does not have a hydroxyl group at position C4, which emphasises the importance of this hydroxyl group (Fig. 12).

## Materials and methods

### Origin of compounds

The dataset of compounds was taken from the COCONUT natural product database (Sorokina et al. 2021) to identify potential lead compounds for future studies. The IUPAC names of compounds 1 through 10 (as seen in Fig. 1) are: (**6**) 5-[(E)-2-(4-hydroxyphenyl)ethenyl]benzene-1,3-diol, (**7**) 5-(2-phenylethenyl)benzene-1,3-diol, (**8**) 5-[2-(4-hydroxyphenyl)ethyl]benzene-1,3-diol, (**9**) [1,1'-biphenyl]-3,4',5-triol, (**10**) 4-[2-(3,5-dihydroxyphenyl)ethenyl]benzene-1,3-diol, (**11**) {3-hydroxy-5-[2-(4-hydroxyphenyl)ethenyl]phenyl}oxidanesulfonic acid, (**12**) 4-[2-(3-hydroxyphenyl)ethyl]benzene-1,3-diol, (**13**) {4-[2-(3,5-dihydroxyphenyl)ethenyl]phenyl}oxidanesulfonic acid, (**14**) 5-(2-phenylethyl)benzene-1,3-diol, (**15**) 5-[2-(3-hydroxyphenyl)ethyl]benzene-1,3-diol.

### Molecular docking study

Molecular docking analysis was performed using the Autodock Vina v.1.2.0 (The Scripps Research Institute, La Jolla, CA, USA) docking software (Trott and Olson 2010). The receptor site was predicted using LigandScout 4.4.8 (Inte: Ligand) Advanced software. The receptor site was predicted using LigandScout 4.4.8 (Inte: Ligand) Advanced software (evaluation license key: 81,809,629,175,371,877,209) (Wolber and Langer 2005), which identified putative binding pockets by creating a grid surface and calculating the buriedness value of each grid point on the surface. The resulting pocket grid consisted of several clusters of grid points, rendered using an iso surface connecting the grid points. The iso surface represents space that may be suitable for creating a pocket. The x-ray crystal structure of the first protein used for docking had the PDB ID: 2V54 (Caillat et al. 2008), which is that of the thymidylate kinase of the vaccinia virus. The second protein crystal structure used for docking analysis was that of the profilin-like protein A42R, and the second was that of the scaffold protein D13 of the vaccinia virus with the PDB ID: 6BED (Garriga et al. 2018). The box center and size coordinates for (PDB ID: 2V54) were 5.6 × 19.2 × 30.6 and 26.2 × 33.2 × 19.9; for (PDB ID: 6BED) was 106.0 × 100.4 × 14.0 and 27.9 × 38.6 × 96.4 around the active site previously predicted by us in Preet et al. (2022). All coordinates used Angstrom units. All ligand and protein structures were prepared using the Dock Prep tool in Chimera 1.16 with default parameters, and the net charges of all ligands were set to neutral(Pettersen et al. 2004).

The following search parameters were used: the number of binding modes was 10, exhaustiveness was 8, and the maximum energy difference was 3 kcal/mol. Results were tested for convergence at exhaustiveness 16 and 24, keeping all the methods mentioned above the same.

#### Validation of molecular docking protocol

**Fig. 13:**
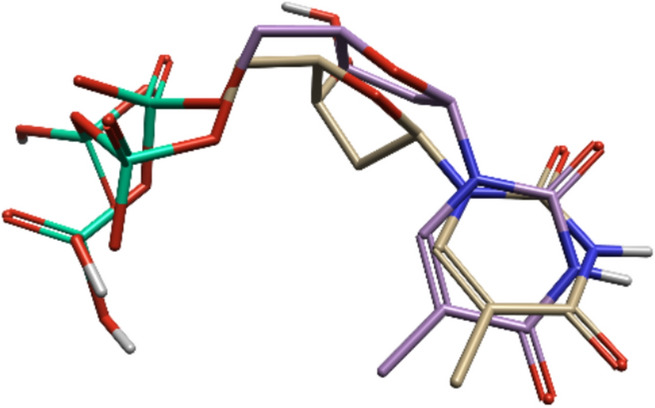
Validation of the docking method by superimposing the co-crystallized ligand (TDP) (purple) and the re-docked ligand (yellow)

The molecular docking protocol was validated using the co-crystallised ligand in the crystal structure of the thymidylate kinase, thymidine diphosphate (TDP). It involves separating the ligand and redocking it, followed by the superimposition of the best-docked conformer to measure the RMSD. The low RMSD of 2.0 Å between the experimental and reference conformations of the ligands indicated that the conformations were the same, validating the docking protocol (Fig. 13).

### Pharmacophore evaluation

LigandScout (Inte: Ligand) Advanced software (evaluation licence key: 44,427,459,425,915,253,797) (Wolber and Langer 2005) was used to generate a 3D pharmacophore model. Default conformation generation settings were used where a maximum number of conformations was 50, timeout (sec) was 600, RMS threshold was 0.8, energy window was 20.0, maximum pool size was 4000, maximum fragment build time was 30, slave memory was set to -1.0 and the number of agent processes was set to 2. The espresso algorithm was used to generate a ligand-based pharmacophore. The generated pharmacophore model compatibility with the pharmacophore hypothesis was created using default settings for LigandScout. Relative Pharmacophore-Fit scoring function with merged feature pharmacophore type and feature tolerance scale factor was set to 1.0 for Ligand-Based Pharmacophore creation. The number of omitted features for the merged pharmacophore was set to 4, partially matching features optional, the threshold (%) was set to 10, and the maximum number of resulting pharmacophores was set to 10. The best model was selected from the ten generated models.

### Molecular dynamics

Molecular Dynamics simulations were performed in Flare version 7.0.0 based on the OpenMM package at 298 K and 1 bar. Supported small molecule force fields are AMBER/GAFF, AMBER/GAFF2, and Open Force Field. The force field used for the protein is AMBER FF14SB. Please refer to the original publication (Eastman et al. 2017) and the OpenMM online documentation for more details on the method. The calculation method used was OpenFF with explicit water, and the simulation length was 200 ns in steps of 4 fs each, where the equilibration was carried out for 200 ps. The solvent model used was the explicit TIP3P water with the shape being a truncated octahedron, and the charge method used was AM1-BCC.

### ADMET study

SMILES notations were inputted into web servers to create ADMET profiles. By using ADMET Lab 2.0 (Xiong et al. 2021) to assess the bioavailability and toxicity of the top polyphenol candidates, we ensure a thorough evaluation of their pharmacokinetics, which subsequently aids the drug development process in prioritizing compounds.

## Conclusions

Mpox, a viral disease historically prevalent in Central and Western Africa, particularly in the Democratic Republic of Congo (DRC), has gained global attention due to recent outbreaks fuelled by increased globalisation. This underscores the urgent need for effective therapeutic interventions. Resveratrol, a natural polyphenolic compound with demonstrated antiviral properties (Cao et al. 2017), has emerged as a potential candidate for anti-mpox drug development. In this study, nine resveratrol analogues were evaluated against key poxvirus proteins using integrated computational approaches, including molecular docking, pharmacophore modelling, and molecular dynamics (MD) simulations. Among these, compound **11** stood out as the most promising candidate, exhibiting a strong docking score, high binding affinity, and stable complex formation with a protein critical for poxviral DNA synthesis. Compound **9** also showed potential, albeit with lower stability in its protein–ligand complex. MD trajectory analyses, encompassing radius of gyration (Rg), hydrogen bond interactions, MM-GBSA binding free energy, and principal component analysis (PCA), further confirmed the superior stability of compound **11**. Additionally, in silico ADMET predictions revealed that the top compounds possess low mutagenicity, moderate to high clearance rates, and low blood–brain barrier (BBB) permeability. Structure–activity relationship (SAR) studies highlighted the significance of hydroxyl groups at positions 4 and 3' for enhancing antiviral activity. This study lays the groundwork for identifying polyphenol-based candidates with anti-poxviral potential, with future research aimed at in vitro validation of the lead compounds. 

The authors emphasise that the increasing frequency of epidemics and pandemics, such as COVID-19 and mpox, underscores the critical need for accelerated antiviral drug development. Effective therapeutics are essential to combat zoonotic diseases, including monkeypox and other poxviruses, which pose a growing threat to global public health.

## Supplementary Information

Below is the link to the electronic supplementary material.

Supplementary file 1 (DOCX 79 KB)

## Data Availability

Data is provided within the manuscript or supplementary information files.
