# Supplementary material for: Computational repurposing of polyphenols for anti-Mpoxviral activity
Source: In Silico Pharmacol. 2025 Apr 17;13(2):65. doi: 10.1007/s40203-025-00345-1 (PMC12006622; doi:10.1007/s40203-025-00345-1)
Supplement: Supplementary file 1 — (DOCX 79 KB) [file 40203_2025_345_MOESM1_ESM.docx]

**Table S1**: COCONUT ID, SMILES, and structures of the compounds studied.

| **COCONUT ID** | **SMILES** | **Structure** |
| --- | --- | --- |
| RVR(CNP0247661) (Standard) | OC1=CC(O)=CC(/C=C/C2=CC=C(O)C=C2)=C1 |  |
| **7** (CNP0359222) | OC1=CC(O)=CC(/C=C/C2=CC=CC=C2)=C1 |  |
| **8** (CNP0200131) | OC1=CC(O)=CC(CCC2=CC=C(O)C=C2)=C1 |  |
| **9** (CNP0203210) | OC1=CC(O)=CC(C2=CC=C(O)C=C2)=C1 |  |
| **10** (CNP0120440) | OC1=CC=C(/C=C/C2=CC(O)=CC(O)=C2)C(O)=C1 |  |
| **11** (CNP0075894) | O=S(OC1=CC(O)=CC(/C=C/C2=CC=C(O)C=C2)=C1)(O)=O |  |
| **12** (CNP0140838) | OC1=CC(CCC2=CC=C(O)C=C2O)=CC=C1 |  |
| **13** (CNP0277001) | OC1=CC(O)=CC(/C=C/C2=CC=C(OS(=O)(O)=O)C=C2)=C1 |  |
| **14** (CNP0165480) | OC1=CC(O)=CC(CCC2=CC=CC=C2)=C1 |  |
| **15** (CNP0256465) | OC1=CC(CCC2=CC(O)=CC(O)=C2)=CC=C1 |  |
